# Incidental noninvasive diagnosis of isolated persistent left superior vena cava in asymptomatic adults: A case report

**DOI:** 10.1097/MD.0000000000050511

**Published:** 2026-09-04

**Authors:** Dian Zhang, Meifang Pan

**Affiliations:** a Department of Ultrasound, Xiangcheng People’s Hospital, Suzhou, Jiangsu Province, China.

**Keywords:** asymptomatic, coronary sinus, echocardiography, IPLSVC, vascular malformation

## Abstract

**Rationale:**

Isolated persistent left superior vena cava (IPLSVC) is an extremely rare congenital malformation with a low overall prevalence in the general population. Most patients present without typical clinical manifestations, leading to a relatively high rate of missed diagnoses in clinical practice. Owing to abnormal venous drainage, IPLSVC is frequently complicated with lesions of the cardiac conduction system, which may further induce arrhythmias.

**Patient concerns:**

A 37-year-old asymptomatic male patient accidentally discovered IPLSVC through chest echocardiography and computed tomography (CT) scans during a routine physical examination. The patient has no history of cyanosis, breathing difficulties, chest pain, or family history of heart disease.

**Diagnoses:**

Transthoracic echocardiography showed coronary sinus dilatation, and chest CT confirmed the absence of the right superior vena cava and the confluence of the right innominate vein into the left superior vena cava. Combined with imaging findings and clinical manifestations, the patient was diagnosed with IPLSVC.

**Interventions:**

Complete echocardiography and chest CT were performed to confirm the diagnosis, inform the patient of relevant procedural risks, recommend regular follow-up examinations and ambulatory electrocardiogram monitoring, and retain imaging data for subsequent diagnosis and treatment.

**Outcomes:**

The patient experienced no discomfort during follow-up. Regular reexaminations and long-term cardiac monitoring were recommended.

**Lessons:**

Although IPLSVC patients are often asymptomatic in the early stages, it may increase the risk of central venous access, cardiothoracic surgery, and pacemaker implantation. Noninvasive echocardiography can provide a basis for early diagnosis of asymptomatic patients and has certain clinical significance. This case helps clinicians further understand IPLSVC in asymptomatic patients, confirms the screening value of routine cardiac ultrasound, and provides practical references for the diagnosis, follow-up, and clinical management of similar cases.

## 1. Introduction

Persistent left superior vena cava (PLSVC) is a rare vascular anomaly. It is considered the most common form of abnormal venous drainage involving the superior vena cava and also the most common congenital anomaly of the thoracic venous system, with an overall prevalence rate of 0.3% to 0.5% in the population.^[1,2]^ According to the different mechanisms of embryonic development and anatomical characteristics, isolated persistent left superior vena cava (IPLSVC) may exist in 10% to 20% of PLSVC cases, often coexisting with other congenital heart diseases or arrhythmias.^[3,4]^ Nearly half of patients with IPLSVC are complicated with congenital heart diseases such as atrial septal defect and endocardial cushion defect.^[5,6]^ Meanwhile, developmental abnormalities of cardiac conduction tissue also increase the risk of various arrhythmias in such patients.^[7,8]^ With the continuous deepening of research on the anatomy and electrophysiology of the coronary sinus and the popularization of cardiac catheterization therapy, the detection rate of this disease has improved. However, the missed diagnosis rate was still high, and the natural course of IPLSVC was still unclear, requiring further research. We report a case of asymptomatic IPLSVC discovered during a routine physical examination, which involves a detailed echocardiography examination and can provide a basis for early diagnosis of such patients.

## 2. Case presentation

**Figure 1.**
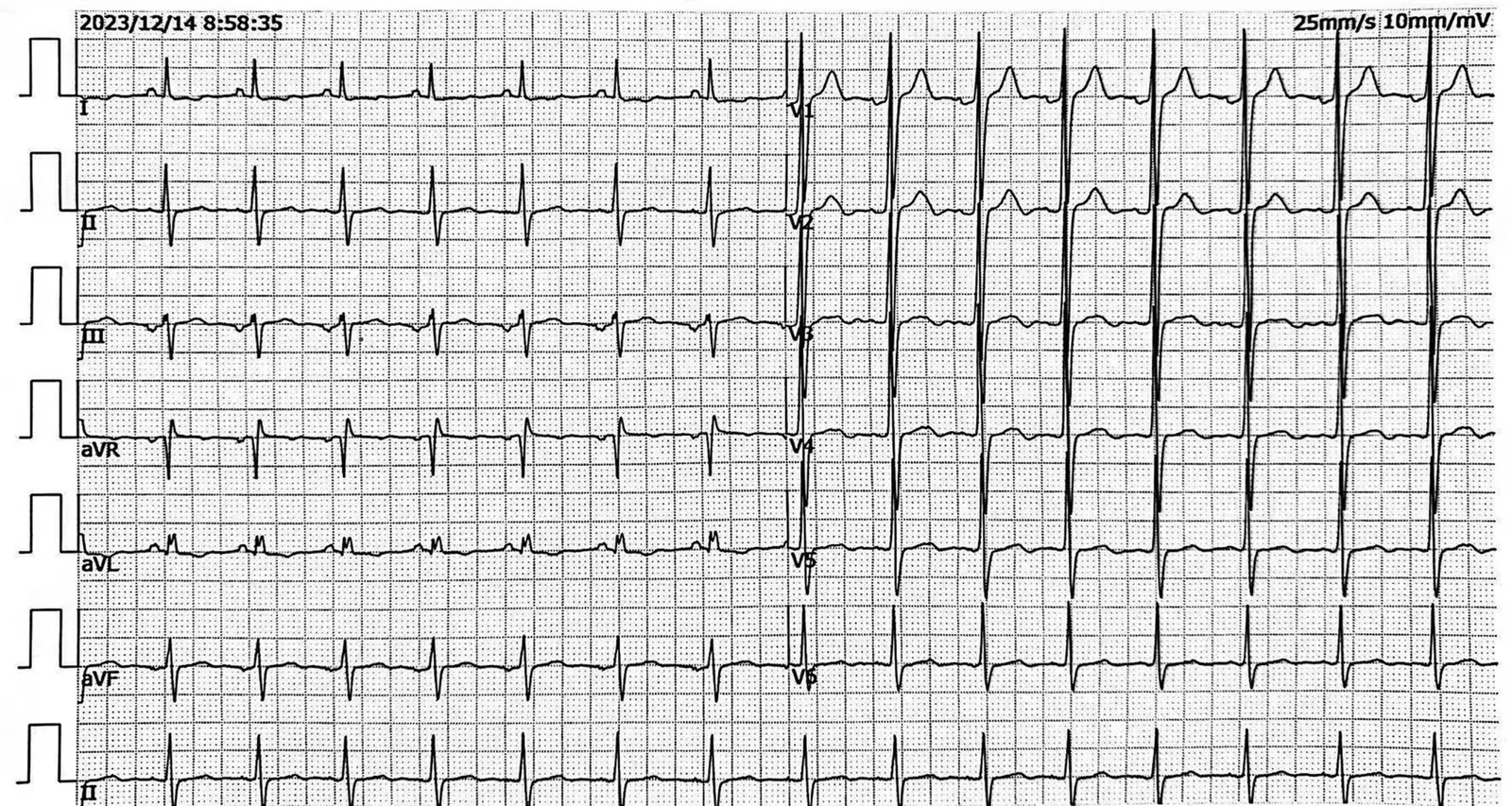
ECG image. The electrocardiogram showed sinus rhythm, short PR interval, and ST-T wave abnormalities.

**Figure 2.**
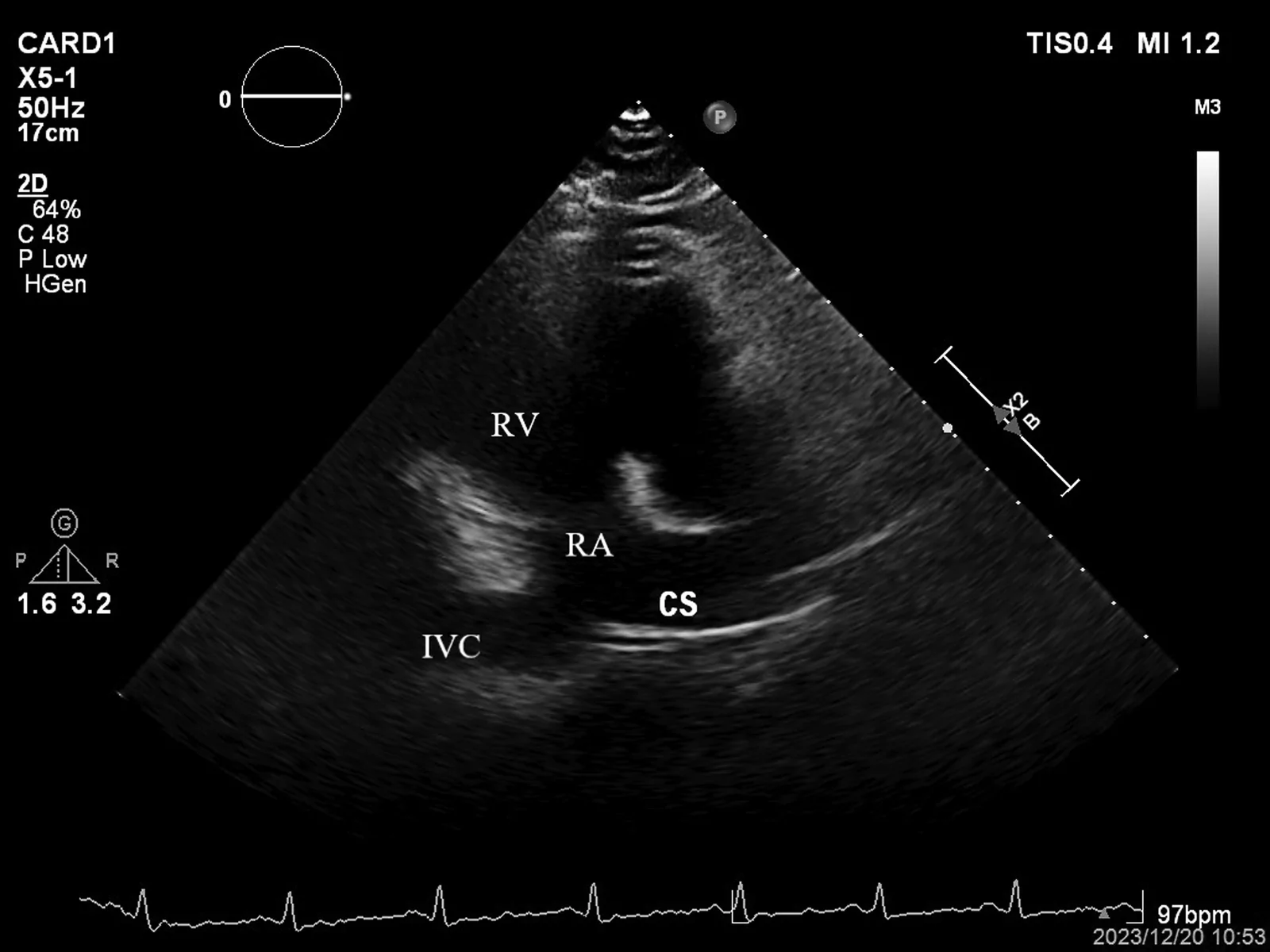
Two-dimensional transthoracic echocardiogram, apical view, showing coronary sinus enlargement (anteroposterior diameter 21 mm). CS = coronary sinus, IVC = inferior vena cava, RA = right atrium, RV = right ventricle.

**Figure 3.**
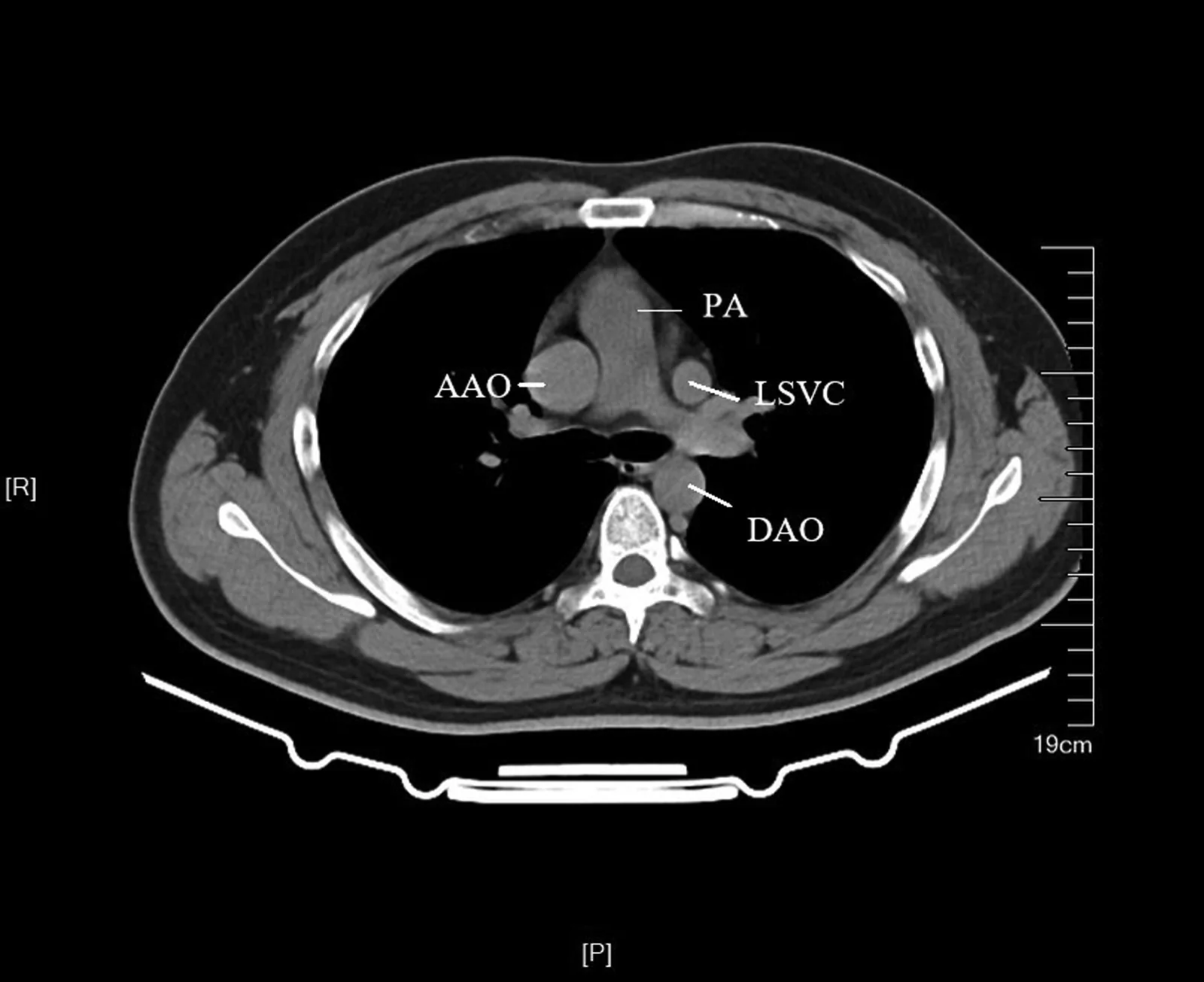
Axial section of CT thorax at (mediastinal window) showing left-sided superior vena cava. AAO = ascending aorta, DAO = descending aorta, LSVC = left superior vena cava, PA = pulmonary artery.

**Figure 4.**
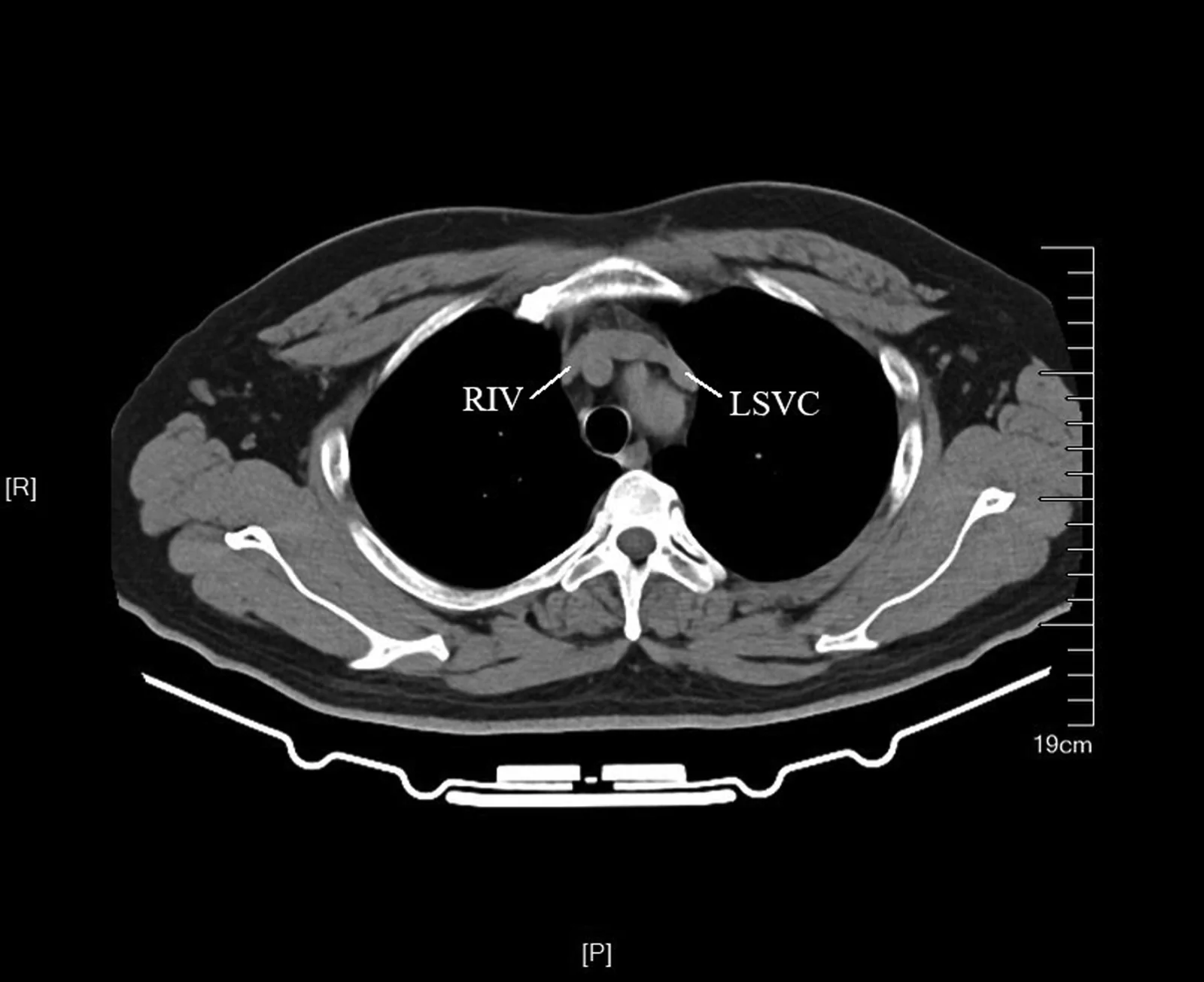
Axial section of CT thorax at (mediastinal window) showing no right superior vena cava, but a right innominate vein merging into the left superior vena cava. CT = computed tomography, LSVC = left superior vena cava, RIV = right innominate vein.

A 37-year-old male went to the health management center for a routine physical examination and had no previous symptoms such as difficulty breathing, cough, hemoptysis, palpitations, paroxysmal nocturnal breathing difficulties, heartburn, reflux, or weight loss. No family history of heart disease. During the examination, the patient’s blood pressure was 140/89 mm Hg, blood sample saturation was 99%, and body mass index was 27.3. The electrocardiogram shows a regular sinus rhythm with a PR interval of 110 ms, a QRS complex of 113 ms, and a QT interval of 365 ms (Fig. 1). Chest and cardiovascular system examinations are normal. The laboratory examination is within the normal range, and the abdominal ultrasound examination confirms that the position and structure of internal organs are normal. A detailed cardiac examination ruled out the existence of any related heart disease. Two-dimensional transthoracic echocardiography (TTE) shows that each atrioventricular cavity is not large, and the thickening of the basal to apical segments of the interventricular septum reaches 12; no significant abnormalities were observed in the amplitude of ventricular wall activity during the resting state; no significant abnormalities were observed in the morphology and activity of each heart valve; color Doppler shows no obvious reflux in each valve; pulse Doppler measurement of diastolic mitral valve orifice blood flow: E peak > A peak; the ejection fraction is 64%; coronary sinus dilation with an inner diameter of approximately 21, with a descending vein visible on the left side of the descending aorta; the right superior vena cava was not explored (Fig. 2). CT scans show no right superior vena cava, but a right innominate vein merging into the left superior vena cava (Figs. 3 and 4). The diagnosis of IPLSVC could be made.

## 3. Discussion

IPLSVC is a rare congenital vascular malformation caused by incomplete regression of the proximal left anterior cardinal vein during embryonic development.^[9]^ Most anomalous veins drain into the coronary sinus or directly enter the right atrium without obvious hemodynamic disturbances, and patients can remain asymptomatic for life, yet it poses prominent risks during invasive procedures. Improper puncture in central venous catheterization may lead to injuries of the mediastinum, pericardium, and pleural cavity.^[10]^ Stenosis of the coronary sinus ostium hinders the placement of pacemaker and defibrillator leads.^[11,12]^ A dilated coronary sinus stretches the His bundle and predisposes patients to malignant arrhythmias including atrial fibrillation and ventricular fibrillation.^[13]^ Frequently combined with atrial septal defect, this malformation increases the risks of air embolism, thromboembolism, and stroke,^[11]^ and also disturbs myocardial perfusion during coronary artery bypass grafting and impairs surgical outcomes.^[14]^ Early identification of IPLSVC helps clinicians assess preoperative risks, adjust surgical plans, and conduct long-term arrhythmia follow-up. This paper reports an asymptomatic case to explore the clinical value of routine noninvasive examinations in its early diagnosis and follow-up.

Current diagnostic methods for IPLSVC include TTE, transesophageal echocardiography, venography, computed tomography (CT), and cardiac magnetic resonance imaging. Featuring noninvasiveness and easy manipulation, TTE is the primary test for PLSVC. Transesophageal echocardiography offers higher sensitivity for PLSVC and concomitant cardiac defects such as atrial septal defect. Chest CT can tentatively diagnose the anomaly but fails to show complete vascular courses,^[15]^ while cardiac magnetic resonance imaging better delineates mediastinal vascular structures.^[16]^ Venography clearly presents venous flow paths, detects tiny intracardiac shunts, and guides device implantation preoperatively.^[17]^ Multimodal combined imaging is suggested for complex multiple venous malformations.^[18,19]^ In this case, TTE found coronary sinus dilation and an aberrant vein beside the descending aorta, and chest CT confirmed right superior vena cava absence with right brachiocephalic vein draining into left PLSVC, finally confirming IPLSVC.

The vast majority of IPLSVC returns venous blood to the coronary sinus, and coronary sinus dilation acts as an indirect sign suggestive of this malformation. Nevertheless, coronary sinus dilation is a nonspecific imaging sign that can be induced by diverse cardiovascular diseases and vascular malformations, mandating comprehensive differential diagnosis to avoid misdiagnosis.^[20]^ In total anomalous pulmonary venous connection, all pulmonary veins drain abnormally into the systemic venous system or right atrium, with echocardiography typically revealing marked dilation of the right ventricle and right atrium alongside abnormal abdominal venous flow direction.^[21]^ Normal cardiac chamber dimensions and unremarkable abdominal venous Doppler signals in this patient ruled out this disease. Coronary arteriovenous fistula is another cause of coronary sinus dilation: abnormal shunting between coronary arteries and the coronary sinus augments coronary sinus blood flow, and severe cases may present with cardiac murmurs and cardiomegaly. No abnormal fistulous tracts or shunt signals were detected on color Doppler in our patient, excluding coronary arteriovenous fistula.^[22]^ Coronary sinus-type atrial septal defect also constitutes a key differential diagnosis; abnormal communication between the left and right atria via the coronary sinus overloads coronary sinus capacitance, with corresponding hemodynamic changes visible on imaging, none of which were identified in this case.^[23]^ In addition, coronary sinus dilation can arise secondarily from functional disorders, predominantly right heart dysfunction, elevated right atrial pressure, and severe pulmonary hypertension. These conditions raise systemic venous pressure and subsequently induce secondary coronary sinus distension.^[11]^ Normal cardiac systolic function, pulmonary artery pressure, and intracardiac pressures in our patient eliminated functional dilation as the underlying cause.

This patient had negligible hemodynamic disturbance given direct venous drainage into the right atrium, without obvious cyanosis. Serial scanning via high intercostal parasternal windows demonstrated coronary sinus dilation. For asymptomatic patients with ultrasound-detected coronary sinus dilation, rigorous differential diagnosis is mandatory. Assessment should not rely solely on the suprasternal window; multiplanar and multi-angle visualization of the anatomical relationships between blood vessels, coronary sinus, and bilateral atria is essential to prevent missed and erroneous diagnoses. Color Doppler and hemodynamic parameters can be integrated for comprehensive interpretation when concomitant cardiac malformations exist. Combined use of contrast echocardiography and multi-slice spiral CT substantially improves detection rates of malformations. We recommended further contrast echocardiography for definitive diagnosis in this patient, yet the patient declined this additional invasive examination due to personal concerns. We fully informed the patient about the congenital vascular anomaly and its potential complications, and advised periodic reexamination and long-term follow-up. Regrettably, follow-up data including 24-hour Holter and cardiac imaging have not been obtained so far, and we will continue to monitor the patient’s condition. Previously published case reports and clinical studies mostly focus on PLSVC patients complicated with congenital heart disease, arrhythmias, overt hemodynamic abnormalities, or incidental intraoperative findings.^[24–26]^ By contrast, our case was a pure isolated IPLSVC free of intracardiac structural lesions, arrhythmias, cyanosis, and hemodynamic disturbances. The combined noninvasive diagnosis strategy of echocardiography plus chest CT was successfully implemented. This typical asymptomatic isolated case deepens clinicians’ understanding of silent IPLSVC, highlights the screening value of routine cardiac ultrasound, and provides practical references for diagnosis, follow-up, and clinical management of similar cases.

## 4. Limitations

This case report has several limitations. Long-term follow-up imaging and Holter data were unavailable to monitor subsequent arrhythmia risks. The patient declined contrast echocardiography, weakening imaging evidence. Moreover, it is only a retrospective single-case study without large-cohort data to calculate the incidence and long-term adverse event rate of asymptomatic IPLSVC.

## 5. Conclusion

Noninvasive echocardiography can provide a basis for early diagnosis of asymptomatic patients. This case helps clinicians further understand IPLSVC in asymptomatic patients, confirms the screening value of routine cardiac ultrasound, and provides practical references for the diagnosis, follow-up, and clinical management of similar cases.

## Author contributions

**Conceptualization:** Dian Zhang.

**Data curation:** Dian Zhang.

**Supervision:** Meifang Pan.

**Visualization:** Meifang Pan.

**Writing – original draft:** Dian Zhang.

**Writing – review & editing:** Dian Zhang.
